# A Programmable Nanofabrication Method for Complex 3D Meta-Atom Array Based on Focused-Ion-Beam Stress-Induced Deformation Effect

**DOI:** 10.3390/mi11010095

**Published:** 2020-01-16

**Authors:** Xiaoyu Chen, Yuyu Xia, Yifei Mao, Yun Huang, Jia Zhu, Jun Xu, Rui Zhu, Lei Shi, Wengang Wu

**Affiliations:** 1 National Key Laboratory of Micro/Nano Fabrication Technology, Institue of Microelectronics, Peking University, Beijing 100871, China; 2 Department of Physics, Key Laboratory of Micro- and Nano-Photonic Structures (MOE), and State Key Laboratory of Surface Physics, Fudan University, Shanghai 200433, China; 3 State Key Lab for Mesoscopic Physics and School of Physics, Peking University, Beijing 100871, China; 4 Electron Microscopy Laboratory, Peking University, Beijing 100871, China

**Keywords:** FIB-SID, 3D nanofabrication, 3D meta-atom array, CL imaging, FTIR spectroscopy

## Abstract

Due to their unique electromagnetic properties, meta-atom arrays have always been a hotspot to realize all kinds of particular functions, and the research on meta-atom structure has extended from two-dimensions (2D) to three-dimensions (3D) in recent years. With the continuous pursuit of complex 3D meta-atom arrays, the increasing demand for more efficient and more precise nanofabrication methods has encountered challenges. To explore better fabrication methods, we presented a programmable nanofabrication method for a complex 3D meta-atom array based on focused-ion-beam stress-induced deformation (FIB-SID) effect and designed a distinctive nanostructure array composed of periodic 3D meta-atoms to demonstrate the presented method. After successful fabrication of the designed 3D meta-atom arrays, measurements were conducted to investigate the electric/magnetic field properties and infrared spectral characteristics using scanning cathodoluminescence (CL) microscopic imaging and Fourier transform infrared (FTIR) spectroscopy, which revealed a certain excitation mode induced by polarized incident IR light near 8 μm. Besides the programmability for complex 3D meta-atoms and wide applicability of materials, a more significant advantage of the method is that a large-scale array composed of complex 3D meta-atoms can be processed in a quasi-parallel way, which improves the processing efficiency and the consistency of unit cells dramatically.

## 1. Introduction

Throughout all ages of human development, all kinds of novel functional materials have been pursued, and every innovation in materials has made a significant contribution to the progress of society. A wide variety of materials with distinctive properties have been gradually developed and applied in various fields. Among all emerging functional materials, the materials that exhibit unique properties in terms of their interaction with electromagnetic waves have been attracting irresistible attention because of the significance and wide application of electromagnetic waves in real life. Most of these materials have unique electromagnetic characteristics in a particular frequency range, such as visible or infrared light, and are usually employed to modulate or manipulate the light in that specific frequency range, so as to achieve certain electromagnetic functions. However, traditional natural materials have encountered increasing challenges with the growing demands for specific electromagnetic properties. As a result, metamaterials/metasurfaces, a kind of artificially engineered material, have been proposed and investigated in the last several decades to realize some extraordinary electromagnetic properties beyond natural materials such as negative refraction, negative permeability, chirality, diffraction-free propagation, and structural color [1,2,3,4].

As an artificial structure, the metamaterials/metasurfaces are usually designed to be a specially arranged, mostly periodic or gradient, meta-atom array of metal or dielectric [5,6]. The meta-atoms that form a metamaterial/metasurface are usually comprised of two-dimensional (2D) or three-dimensional (3D) subwavelength structure unit cells which show certain particular electromagnetic properties in a specific frequency range [7,8,9]. In addition to designing various meta-atom unit structures to achieve specific functions, the fabrication of structures is also an extremely critical aspect, otherwise, even the best design could be just a castle in the air. With the deepening and expansion of metamaterial research, 2D nanostructures are not enough to meet all requirements. Thus, 3D nanostructures represent inevitable solutions to realize some special functions [10]. Nevertheless, different from the fabrication of simple 2D structures, the fabrication of complex 3D nanostructures array faces much greater challenges despite the rapid development of 3D nanofabrication in recent years [11,12,13].

As shown in Table 1, several typical fabrication methods for 3D micro/nanostructure, including direct laser writing (DLW) [14,15,16,17,18], self-assembly [19,20], and nanoimprint lithography [21,22,23,24] have been widely used in specific fields. Although the mentioned methods have their own unique advantages, some disadvantages still exist, which limit their further application in 3D micro/nanostructure array fabrication. Direct laser writing, as a micro/nanofabrication method based on laser-assisted and photo-polymerization, has a distinctive ability to fabricate complex 3D structures with nanometer-scale precision but faces efficiency problems when fabricating a large-scale structure. Self-assembly is a common method of 3D micro/nanofabrication and the basic principle is to transform the pre-defined 2D pattern into target 3D structure by a certain driving force such as surface tension, electrostatic force, magnetic force, thermal deformation and so on. Despite the diverse ways of implementing self-assembly fabrication, they are usually only suitable for specific situations. Nanoimprint lithography, a patterning process to transfer a designed master mold into the resist, can process micro/nanostructures with both high throughput and low cost, however, the processing difficulty increases rapidly with the complexity of the 3D structure.

Focused ion beam (FIB) is a widely used technology in the process of milling, imaging, and deposition by focusing and accelerating charged ion beams (gallium ions most commonly used) onto materials [25,26,27]. Apart from the conventional functions in fabrication, FIB has also been developed to conduct some other fabrication techniques with its unique advantages in recent years, among which the FIB stress-induced deformation (FIB-SID) effect is one of latest developing techniques applied in micro/nanofabrication. The most attractive aspect of this FIB-SID technique is the capability of bending suspended thin films of various materials in both directions (−70–90°) precisely by controlling the ion dose and irradiation area of FIB and, the deformation might be caused by the stress change in the films under FIB irradiation [28]. The FIB-SID technique is a newly developed method to fabricate complex 3D micro/nanostructures [29,30,31,32,33], which can be used in optical modulation, controllable drug delivery, cell capsulation, and many other areas [34].

Compared to other 3D nanostructure array fabrication methods, the FIB-SID technique can process, in real-time, complex 3D structures with nanoscale precision and is versatile for diverse materials [28,35]. However, when the scale of processing becomes large, the processing efficiency of this method remains a non-negligible challenge because of its serial processing mode, which means, as reported in our previous work [28,35], the 3D meta-atoms can only be fabricated one by one unit cell.

In this paper, we present, for the first time, a novel programmable 3D nanofabrication method based on the FIB-SID technique, which is capable of fabricating a complex 3D meta-atom array of large scale in a quasi-parallel way on a suspended thin film. To demonstrate this method, we designed a kind of 3D meta-atom array and fabricated a batch of the arrays using the presented method. Cathodoluminescence (CL) imaging and Fourier transform infrared (FTIR) spectroscopy were also conducted to analyze the electromagnetic characteristics of the proposed 3D meta-atom arrays.

## 2. Structure Design

### 2.1. 3D Meta-Atom Structure Design and Unfolding Pattern Figure

**Figure 1:**
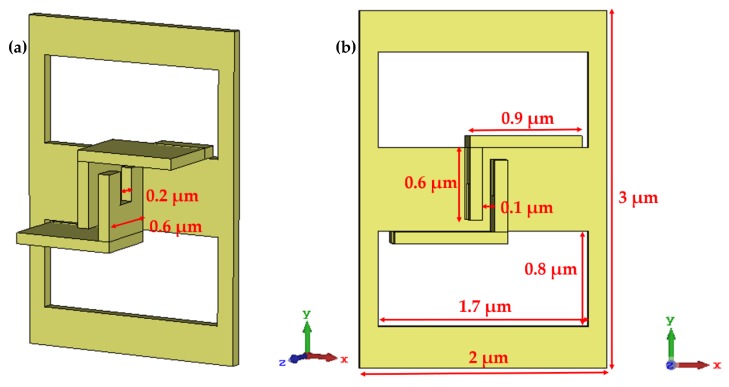
The designed structure of 3D meta-atom. (**a**) Oblique and (**b**) overhead views of a 3D meta-atom unit cell, which contains a pair of coupled claws with a gap of 0.1 μm, a length of 0.9 μm and a height of 0.6 μm. The whole array is 90 μm by 60 μm with a period of 3 μm in Y-axis direction and 2 μm in X-axis direction.

As mentioned above, 3D micro/nanostructures fabricated by FIB-SID technique can be used in many optical and biological areas. An important optical application is to realize narrow band or broadband absorption by generating strong electromagnetic resonance inside the 3D nanocavities or nanogaps [31,36]. Here, we design a 3D meta-atom structure that contains a pair of double claw structure and two rectangle holes in a single meta-atom (Figure 1), which contains both vertical and horizontal gaps to ensure the generation of strong electromagnetic resonance. It is not easy to fabricate such an array composed of the designed complex 3D meta-atom structure with high efficiency and high quality by conventional methods. This is why the unique advantage of the FIB-SID technique comes into play. Therefore, the fabrication of such a 3D meta-atom array can be a demonstration of the newly proposed FIB-SID based programmable quasi-parallel 3D nanofabrication method.

**Figure 2:**
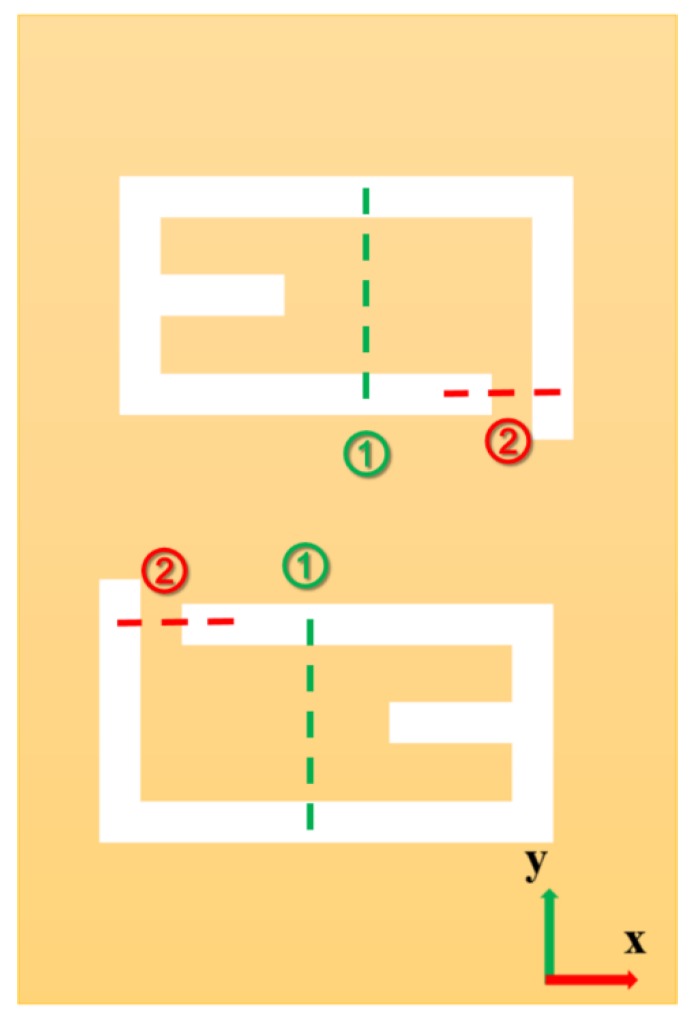
The unfolding 2D pattern figure of the designed 3D unit cell, which is the initial state derived reversely from the forming process of 3D meta-atom by FIB-SID bending. The FIB-SID bending areas for transformation processes are marked as green lines and red lines, respectively.

To fabricate a complex 3D meta-atom array using the FIB-SID technique on a suspended thin film, the corresponding unfolding 2D pattern should be acquired first. Then, the transformation figures, which specify the areas where the FIB-SID technique will be applied to transform 2D patterns into a 3D structure, can be drawn according to the designed 3D structure. The transformation process can be conducted more than once to fabricate some complex 3D meta-atom structures which also means the drawing of more than one transformation figure. The key to fabricating a complex 3D structure array is the design of appropriate transform figures. Using this method, a 3D meta-atom array can be processed at one time with transformation figures. In this coupled claw-shaped design, we analyzed the bending process of the structure and drew a schematic to show the corresponding unfolding 2D pattern and bending areas for 3D transformations of the unit cell, as shown in Figure 2.

### 2.2. Numerical Simulation of the 3D Meta-Atom Arrays

The 3D meta-atom arrays are comprised of periodic nanostructures that contain a pair of coupled claw-shaped structures in each unit cell. A series of numerical simulations of the electromagnetic field was carried out using the finite element method to figure out how IR light interacted with the structure theoretically. Strong electromagnetic resonance could be generated inside the claw gaps, realizing distinct wavelength-selective absorption in the infrared band.

**Figure 3:**
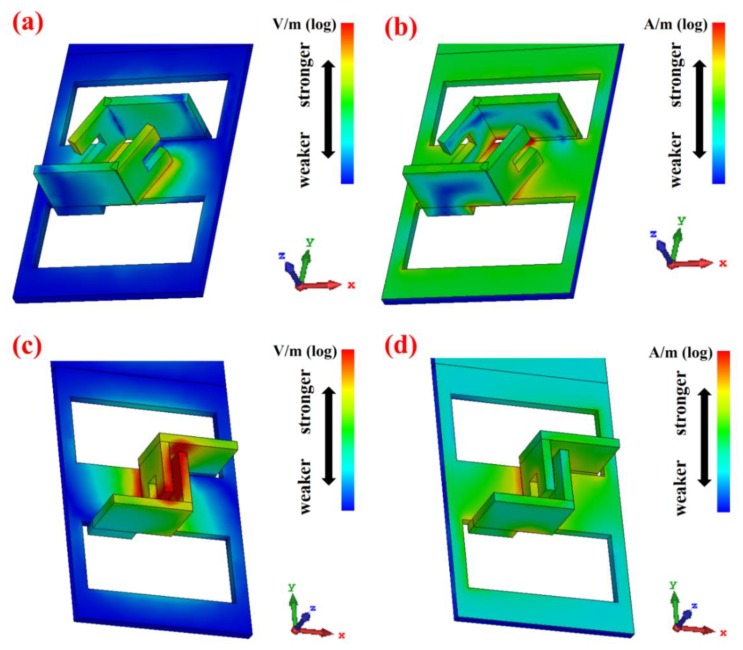
The electric/magnetic field distribution of 3D meta-atom unit cells with different claw gap distance. (**a**) Electric field and (**b**) magnetic field distribution simulation results of the 3D meta-atom array with larger claw gap, (**c**) electric field and (**d**) magnetic field distribution simulation results of the 3D meta-atom array with smaller claw gap.

Figure 3 shows the visualized electric/magnetic field distribution of a meta-atom unit cell in the array under normally incident IR light with a linear polarization direction of the X-axis (λ = 8.5 μm). The simulations were conducted for two meta-atom arrays with the same size but different gaps between coupled claws, respectively. The geometric dimensions of the meta-atom with a smaller claw gap (Figure 3c,d) used in the simulations are shown in Figure 1. The S-parameters were also extracted from the simulation results.

Referring to the simulation results shown in Figure 3, the red color indicates a stronger electric/magnetic field while the blue color means weaker distribution in the area. It is clear that electric resonance becomes much stronger around the claw when the claw gap gets smaller. This remarkable distinction might indicate some significant properties of the 3D meta-atom array at the IR wavelength.

**Figure 4:**
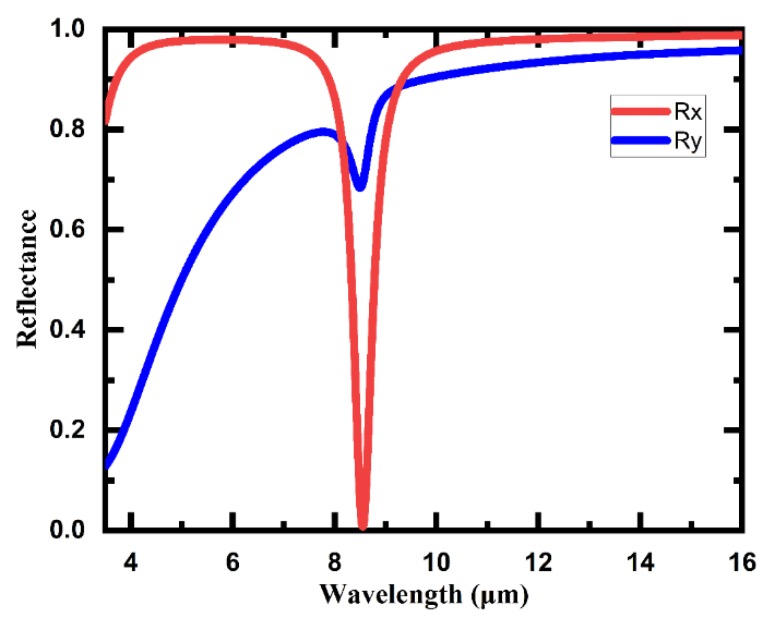
Simulated reflection spectra of the 3D meta-atom array with a smaller claw gap. Rx and Ry are the reflection spectra of the array under normally incident IR light with the polarization direction parallel to X-axis and Y-axis, respectively.

Then, we went further to calculate reflection spectra from the S parameters extracted from electric/magnetic field simulation results of the 3D meta-atom array with a smaller gap. The light source was normally incident IR light, linearly polarized, from 3.5 μm to 16 μm, and the reflection spectra of both polarized directions of the X-axis (Rx) and Y-axis (Ry) were calculated, as shown in Figure 4. The reflection spectra, Rx and Ry, show significant differences. In terms of reflectance, the spectrum Rx remains high except for a sharp dip identified at around 8.5 μm. On the contrary, the spectrum Ry decreases rapidly as the wavelength of incident light decreases except a small dip at around 8.5 μm. The quality factors, defined as the ratio of resonance frequency to the full width at half maximum, are 15.84 and 18.59 for resonance frequency ω_1_ = 35.1 THz in Rx and ω_2_ = 35.4 THz in Ry, respectively, and the corresponding lifetimes of the resonance are 71.8 fs for ω_1_ and 83.5 fs for ω_2_. These distinctive characteristics in the reflection spectra manifest that some interesting properties of the 3D meta-atom array might remain to be found.

## 3. Fabrication

### 3.1. Preparation of Suspended Gold Films

**Figure 5:**
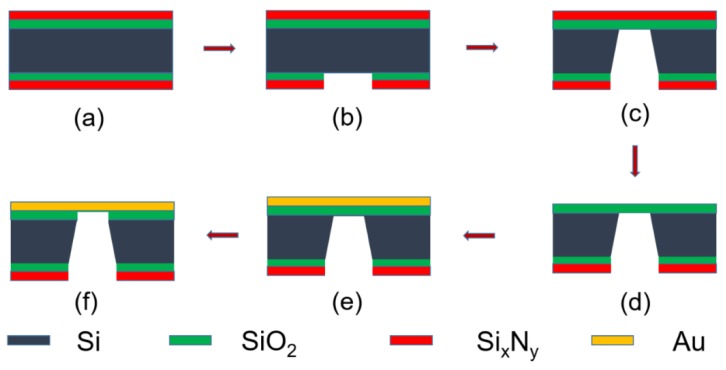
MEMS process diagram of the suspended gold film. (**a**) LPCVD for silicon dioxide and silicon nitride layer on both sides separately, (**b**) Lithography and RIE to expose square windows on the backside, (**c**) Wet etching to remove exposed silicon substrate using KOH solution, (**d**) RIE to remove all the silicon nitride layer on the front side, (**e**) Gold film deposition of 120 nm using PVD process, and (**f**) Release-etching of silicon dioxide layer to complete the process with BHF solution.

To conduct the proposed programmable nanofabrication, a combination of the conventional micro-electromechanical systems (MEMS) process and FIB treatment is usually indispensable for a complex 3D meta-atom array. The FIB treatment is based on a suspended thin film, which is comprised of a single or several layer(s) of metal and/or dielectric. The suspended films are fabricated by the conventional MEMS processes. A typical process of the suspended gold film is shown in Figure 5.

Firstly, a silicon dioxide film (sacrificial layer) and a silicon nitride film (mask layer) were deposited on both sides of a clean silicon substrate successively using low pressure chemical vapor deposition (LPCVD) process (Figure 5a). Then, photolithography and reactive ion etching (RIE) were used to expose a series of square windows on the backside (Figure 5b). The exposed silicon windows were immersed into KOH solution for anisotropic wet etching to remove the silicon substrate (Figure 5c). After that, the silicon nitride film on the front side was removed thoroughly using RIE and a gold film of 120 nm was coated on the surface using magnetron sputtering (Figure 5d,e). Finally, the sacrificial layer (SiO_2_) was corroded with BHF solution and the prepared suspended gold film was ready for the subsequent FIB process (Figure 5f).

### 3.2. Programmable Quasi-Parallel Fabrication Method for 3D Meta-Atom Arrays Using FIB-SID Technique

After all the above preparations have been completed, the FIB process can be carried out. The FIB fabrication process and in-situ scanning electron microscopic (SEM) imaging were both conducted in a FIB dual beam system (FEI strata DB235). The ion beam accelerating voltage of the FIB process was fixed at 30 keV, and the beam current was set to be 100 pA, with a dwell time of 1 μs and an overlap of 50%.

The process of 3D meta-atom array fabrication using the FIB-SID technique can be described in three main steps. Step 1 is to import the prepared unfolding 2D pattern and the transformation figures into FIB system and set proper magnification and FIB process parameters. Step 2 is to fabricate the suspended gold thin film using the FIB milling function so as to obtain the pre-designed unfolding 2D pattern structures. Step 3 is to conduct the FIB-SID treatment on a series of specific areas of the 2D pattern in sequence as the transformation figures indicate.

Here, the FIB process is automatically conducted after the parameters are set. Moreover, the FIB-SID process is conducted in a “quasi-parallel” way instead of in serial so that the whole 3D structure array fabrication could be completed at one time. Compared with our previous serial fabrication method that processes 3D structures one cell at a time, this programmable array fabrication method for complex 3D nanostructures improves the fabrication efficiency to a new level.

**Figure 6:**
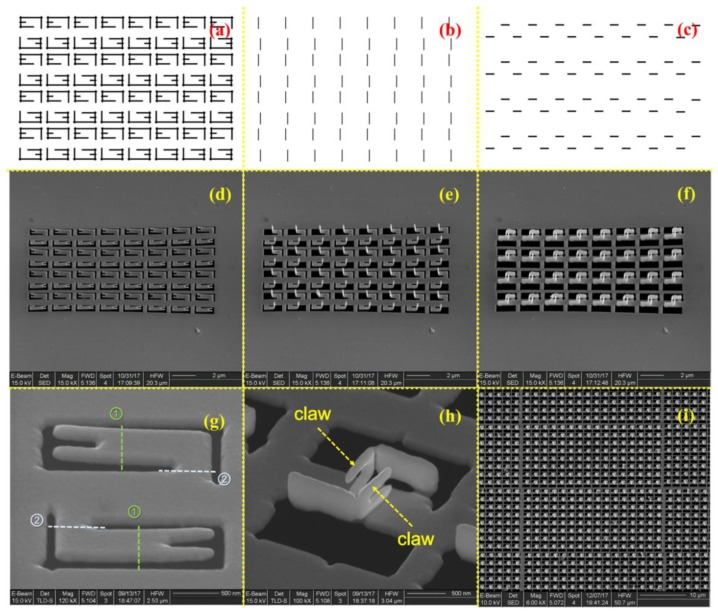
Main fabrication steps and process details of the designed 3D meta-atom array based on the FIB-SID technique. (**a**) unfolding 2D pattern figure and (**b**,**c**) the 3D transformation figures for FIB process, (**d**) unfolding 2D patterning using the FIB milling function to irradiate as (**a**) drawing, (**e**,**f**) 3D transformation process using the FIB-SID technique to irradiate as (**b**,**d**) drawing, (**g**) a unit cell of unfolding 2D patterned structure which marked irradiation area of FIB-SID process as dotted line, (**h**) a unit cell of completed 3D meta-atom array with two coupled claws facing to each other, (**i**) a part of large scale 3D meta-atom array fabricated using this method. The scale bars are 2 μm for (**d**–**f**), 500 nm for (**g**,**h**) and 10 μm for (**i**).

The presented programmable quasi-parallel fabrication method shows both high efficiency and unique programmability for complex 3D meta-atom array fabrication. Figure 6 shows the main fabrication steps of a 3D meta-atom array using this method as a demonstration. To fabricate a complex 3D structure array as designed in Figure 2 on a suspended gold thin film, we first drew the unfolding 2D pattern (Figure 6a) and the transformation figures (Figure 6b,c) of the 3D meta-atom array. Then, the FIB process was conducted and in-situ SEM observation followed the process using the FIB dual beam system (Figure 6d–i).

As shown in Figure 6d, the FIB milling function, usually with a larger FIB dose, was first used to fabricate the unfolding 2D pattern of 3D meta-atom array according to the pre-designed figure (Figure 6a). Figure 6g shows the detailed pre-designed figure of a single unit cell in the 2D pattern array. Figure 6e,f shows the subsequent transformation process of the 3D meta-atom array, in which the FIB-SID technique was utilized to bend each gold suspended cantilever of the 2D pattern according to the pre-drawn transformation figures (Figure 6b,c) in sequence. The bending angle depends on the fabrication parameters during FIB process, including accelerating voltage, beam current, and process time of the ion beam. The dose of the ion beam used in FIB-SID was much smaller than that in the FIB milling process, usually one or two orders of magnitude. The transformation figures indicated the specific irradiating area of the ion beam, and the figure was treated as a whole to conduct FIB-SID process in a quasi-parallel way. The FIB process areas for each unit cell in the two transformation procedures were marked as dotted lines with different colors in Figure 6g. Figure 6h shows the result of one 3D meta-atom unit cell fabricated by this method, in which contains a pair of coupled claw structure. A large scale of this complex 3D meta-atom array could be finally fabricated using this programmable quasi-parallel fabrication method (Figure 6i).

It is also worth noting that the programmable quasi-parallel fabrication process is not limited to the coupled claw-shaped structure proposed in this paper. Other 3D structures based on suspended films could also be easily fabricated by designing different unfolding 2D pattern and corresponding transformation figures. The FIB was applied in two ways in most previous work on FIB-induced bending for 3D nanostructure. One such way is to irradiate ions as a whole without discrimination, which could realize some special fabrication or manipulation [30,37,38,39,40,41], while the other way is to irradiate ions on a specific area in sequence, which could fabricate a 3D array one unit by one unit with low efficiency [31,35,42]. As a comparison, the newly proposed fabrication method in this paper is capable of fabricating complex 3D meta-atom arrays in a quasi-parallel way to process all the unit atoms as a whole, which brings some remarkable advantages, including a much higher efficiency as well as a significant improvement in consistency and yield of the unit atoms. Furthermore, this method retains the advantages of ion-irradiation programmability and material variety in fabricating complex 3D nanostructures.

### 3.3. SEM Images of the So-Fabricated 3D Meta-Atom Arrays

**Figure 7:**
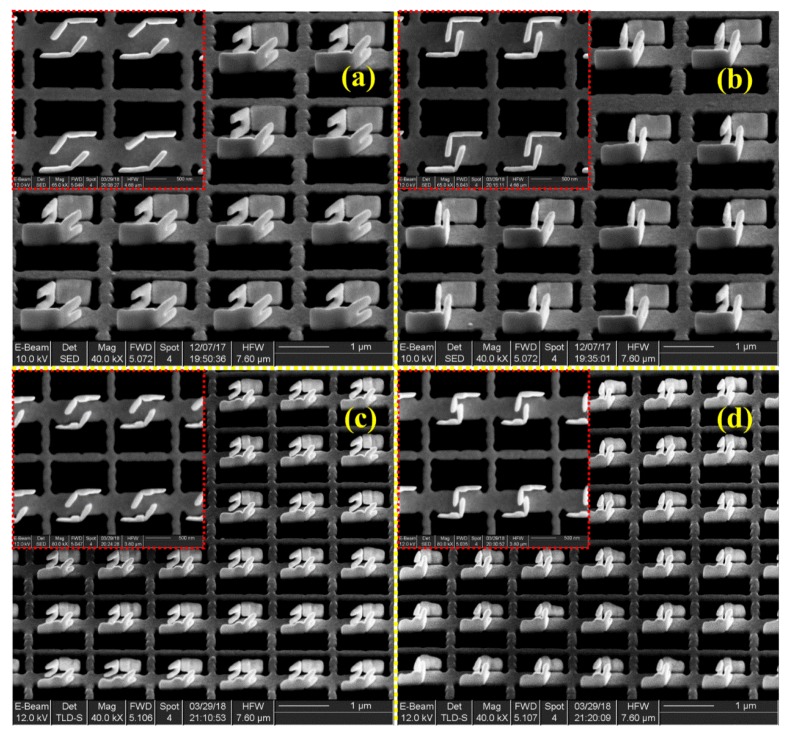
SEM images of the fabricated 3D meta-atom arrays with different cell sizes and claw distances. The inset images are the top view of each 3D meta-atom array, respectively. The overall size is around 60 μm × 90 μm. (**a**,**b**) are arrays of the same size with a larger claw distance of ~500 nm (**a**) and a smaller distance of ~150 nm (**b**), both with a claw length of ~500 nm. (**c**,**d**) are arrays of the scaled down size with larger claw distance of ~250 nm (**c**) and smaller distance of ~70 nm (**d**), both with a claw length of ~330 nm. The scale bars are 1 μm.

Using the proposed programmable quasi-parallel fabrication method, a batch of 3D meta-atom arrays were fabricated. Figure 7 shows the in-situ SEM images. By setting the process parameters of FIB-SID, the bending angle in the transformation could be controlled precisely to fabricate exactly what was designed. Figure 7a (large size with big gaps, marked as Array1) and Figure 7b (large size with small gap marked as Array2) are two 3D meta-atom arrays with the same structure size and period but different gap distance of the coupled claw. Figure 7c,d are scaled-down versions of Figure 7a,b. Here, the four insets show the different claw gaps of 3D meta-atoms from the top view, respectively. These SEM images clearly demonstrate the validity and processing accuracy of the presented FIB-SID array fabrication method. The geometry parameters of actual fabrication are slightly different from those used in simulations.

## 4. Optical Characterization and Discussion

### 4.1. CL Characterization of the 3D Meta-Atom Arrays

In order to analyze the electromagnetic field characteristics of the fabricated 3D meta-atom arrays, scanning CL microscopy was applied to acquire their CL images. As an increasingly significant technique, CL imaging can directly reflect the intensity distribution of light emission from a material excited by incident electrons. The excitation intensity of all regions of the structure can be displayed visually. Moreover, the CL image contains a variety of information generated when the electron beam interacts with the material, which visibly facilitates the direct analysis of various properties of the material, and thus the CL microscopy technique has been widely used in the material characterization field [43].

**Figure 8:**
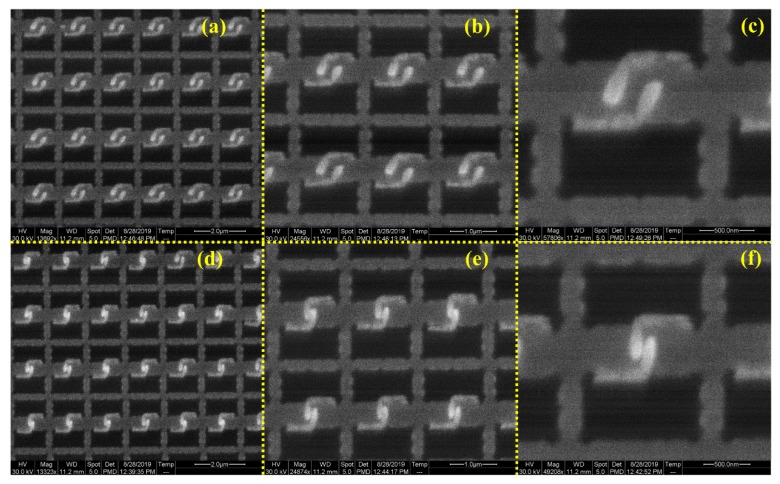
CL (cathodoluminescence) images of the 3D meta-atom arrays with different claw gaps. (**a**–**c**) CL images of the same 3D meta-atom arrays with larger claw gaps in different magnifications, and (**d**–**f**) CL images of the same 3D meta-atom arrays with smaller claw gaps in different magnifications. The scale bars are 2 μm for (**a**,**d**), 1 μm for (**b**,**e**) and 500 nm for (**c**,**f**).

As shown in Figure 8, CL images of the 3D meta-atom arrays were obtained to analyze the electromagnetic field characteristics of excited states. The brighter the area in the CL image, the greater the excitation intensity in the structure. It is clear that the excitation regions notably concentrate on the claws of the 3D meta-atom, forming some periodic, prominent bright spots. These bright spots get much brighter when the gap distances become smaller, which indicates stronger excitation intensity occurred in the 3D meta-atom array with a smaller claw gap.

The CL images are also consistent with the simulation results in Figure 3. In other words, the regions with stronger electromagnetic field distribution are also the same regions with greater excitation intensity, which manifests theoretically and experimentally that the excitation mode of the 3D meta-atom array with smaller claw gap has changed and the interaction between the structure and the incident light has increased.

### 4.2. IR Spectra Characterization of the 3D Meta-Atom Arrays

**Figure 9:**
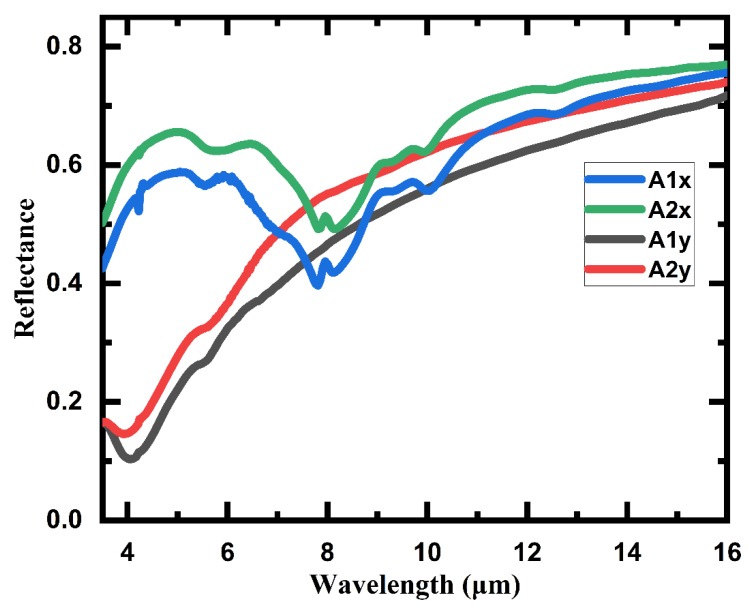
Measured IR reflection spectra of the 3D meta-atom arrays under normal incidence, with the polarization along the X-axis (A1x, A2x) and Y-axis (A1y, A2y). The tested samples are Array1 (A1x, A1y) and Array2 (A2x, A2y).

In order to investigate the IR spectral characteristic of the structure, we also measured the IR reflectance spectra of 3D meta-atom arrays with different gaps (Array1 and Array2) using FTIR spectrometer (Bruker Spectrometer FT-IR Vertex 70 IR Microscope Hyperion 1000, Billerica, MA, USA). Combined with the simulated spectra, the measured spectra could be an intuitive and powerful way to analyze the electromagnetic field characteristics of the 3D meta-atom array. As shown in Figure 9, the IR spectra were measured with linearly polarized IR light under normal incidence, including both the X-axis and Y-axis direction of linearly polarized light for different 3D meta-atom arrays respectively, and the measured wavelength range covered 3 μm to 16 μm.

The profiles of the IR reflectance spectra are basically consistent with the simulation results in Figure 3. The two spectra of 3D meta-atom arrays with different gaps have similar profiles with few differences. On the other hand, the reflectance spectra of linearly polarized light in the X-axis direction are significantly different from that in the Y-axis direction. Here, we take the spectrum of Array2 as an example to specifically analyze the spectral characteristics of the 3D meta-atom array. On the whole, the reflectance of 3D meta-atom arrays decreases as the wavelength decreases for normally incident IR light with a linear polarization direction of the Y-axis. The reflectance decreases even faster in the shorter wavelength region, resulting in notably low reflectance (<50%) for a wavelength of incident light shorter than 7 μm. On the contrary, for incident IR light with a linearly polarized direction of the X-axis, the reflectance spectrum of the 3D meta-atom array remains relatively high in most measured regions, except for a dramatic dip near 8 μm. Combined with the previous analysis of numerical simulations and CL images, the dip in this spectrum indicates that field enhancement around the claws of the 3D meta-atom array may generate near 8 μm, suggesting an occurrence of a certain excitation mode of interaction between incident light and the array, which might be applied in detection and/or sensing fields.

**Figure 10:**
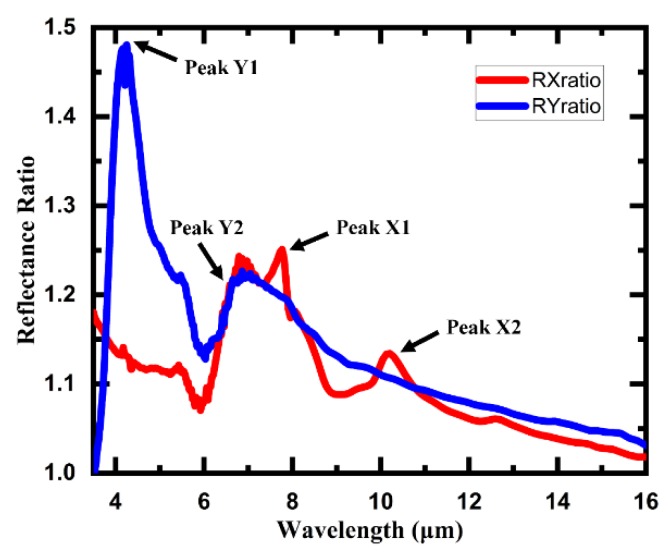
The reflectance ratio spectra of the 3D meta-atom array for the incident light with different polarized directions. RXratio and RYratio describe how much the reflectance of smaller claw gap array (Array2) varies with respect to that of larger claw gap array (Array1) under IR incident light with linear polarization direction of X-axis and Y-axis, respectively.

In addition to the interesting IR properties of the 3D meta-atom arrays, some further spectral characteristics of the structures with different claw gaps are also worthy of further exploration. As shown in Figure 10, the reflectance ratio spectra of the structure for the incident light with different polarized directions were calculated from the IR reflectance spectra in Figure 9, respectively. The calculation formulas are as follows:

(1) $$RXratio=\frac{A2x}{A1x},$$

(2) $$RYratio=\frac{A2y}{A1y}.$$

The formulas are defined simply to reflect how much the reflectance of smaller claw gap array (Array2) varies with respect to that of larger claw gap array (Array1) under IR incident light with linear polarization direction of X-axis and Y-axis, respectively. With regard to the incident light with the linear polarization direction of the X-axis, the RXratio profile shows two remarkable peaks, which indicates that the reflectance of the smaller claw gap array (Array2) is enhanced dramatically compared to that of the larger claw gap array (Array1). The larger peak (peak X1) covers 6 μm to 9 μm and reaches the maximum of 1.25 at 7.8 μm, while the smaller peak (peak X2) lies around 10 μm and reaches the maximum of 1.13 at 10.2 μm. On the other hand, when it comes to the incident light with the linear polarization direction of the Y-axis, the RYratio profile also exhibits two remarkable peaks. The larger peak (peak Y1) of RYratio is even more striking and reaches a maximum of 1.48 at 4.2 μm while the smaller peak (peak Y2) basically overlaps with the peak X1.

These four unique peaks reflect some significant characteristics of the 3D meta-atom array in IR wavelength and might be applied in various fields such as molecular detection and IR polarization sensing. It is well known that only the IR light with some specific wavelengths can pass through the atmosphere with low absorption, including mid-wave (3.5–5 μm) and long-wave (8–14 μm) IR light. Luckily, the peak Y1 and peak X2 are located just within the range of mid-wave IR and long-wave IR, respectively, which indicates that some significant applications might be realized based on these two peaks of the reflectance ratio.

## 5. Conclusions

To conclude, a programmable nanofabrication method for complex 3D meta-atom arrays based on the FIB-SID effect was presented and a distinctive nanostructure array composed of periodic 3D meta-atoms with a coupled-claw in each unit cell was realized. The 3D meta-atom arrays with different claw gaps were designed and fabricated to demonstrate the presented nanofabrication method. To analyze the properties of the designed 3D meta-atom arrays, numerical simulations on electric/magnetic field distribution and IR reflectance spectra of the arrays under normal incident linearly polarized IR light were computed using the finite element method before fabrication. Then, a series of measurements were conducted to investigate the electric/magnetic field properties and IR spectral characteristics of the fabricated 3D meta-atom arrays, including scanning CL microscopic imaging and Fourier transform IR spectroscopy.

The presented nanofabrication method shows several unique advantages for 3D meta-atom arrays based on a suspended thin film. The most significant advantage is that a large-scale array composed of complex 3D meta-atoms can be processed in a quasi-parallel way, which improves the processing efficiency and the consistency of unit cells dramatically. Furthermore, the method is programmable for all kinds of complex 3D structures and is suitable for various materials. Both the numerical simulations of electric/magnetic field distribution and measured CL images of the fabricated 3D meta-atom arrays suggest that a strong excitation can be induced near the claws of the structure under normally incident IR linearly polarized light. The measured reflection spectra of the 3D meta-atom array show distinct polarization dependence. There is a dramatic dip near 8 μm in reflection spectrum of the array under incident IR light with a linearly polarized direction of the X-axis, which reveals a certain excitation mode of interaction between incident light and the array generated near 8 μm. Furthermore, the reflectance ratio spectra of the structure for the incident light with different polarized directions, RXratio and RYratio, were also calculated, in which a striking peak of RXratio and a gentle peak of RYratio are located just within the range of mid-wave IR and long-wave IR windows, respectively.

Based on the measured distinctive spectral characteristics of the designed 3D meta-atom arrays, some applications in the IR region might be developed in the future, such as IR detection, sensing, and modulation. The proposed fabrication method could also be used to fabricate other 3D arrays with high fabrication efficiency and precision.

## Figures and Tables

**Table 1:** Comparison of the four micro/nanofabrication fabrication methods for 3D structures.

Methods	Advantages	Disadvantages
direct laser writing	High precision and suitable for complex structure	Low efficiency for large scale
Self-assembly	Flexible and molecular scale	Limited scope of application
Nanoimprint lithography	high reproducibility and low cost	Hard for complex structure
FIB-SID (Focused ion beam stress-induced deformation)	Large-scale complex 3D structures array with high precision and versatility	Limitation of suspended film based
